# Loss of *Lkb1* cooperates with *Braf^V600E^* and ultraviolet radiation, increasing melanoma multiplicity and neural‐like dedifferentiation

**DOI:** 10.1002/1878-0261.13715

**Published:** 2024-08-08

**Authors:** Kimberley McGrail, Elena González‐Sánchez, Paula Granado‐Martínez, Roberto Orsenigo, Yuxin Ding, Berta Ferrer, Javier Hernández‐Losa, Iván Ortega, Juan Martín‐Caballero, Eva Muñoz‐Couselo, Vicente García‐Patos, Juan A. Recio

**Affiliations:** ^1^ Biomedical Research in Melanoma‐Animal Models and Cancer Laboratory Vall d'Hebron Research Institute VHIR, Vall d'Hebron Hospital‐UAB Barcelona Spain; ^2^ Anatomy Pathology Department Vall d'Hebron Hospital‐UAB Barcelona Spain; ^3^ Animal Laboratory Unit Biomedical Research Park of Barcelona‐PRBB Spain; ^4^ Clinical Oncology Program, Vall d'Hebron Institute of Oncology (VHIO) Vall d'Hebron Hospital‐UAB Barcelona Spain; ^5^ Dermatology Department Vall d'Hebron Hospital‐UAB Barcelona Spain; ^6^ Present address: Miltenyi Biotec S.L. Madrid Spain; ^7^ Present address: University of Barcelona Bellvitge Spain; ^8^ Present address: Instituto Cajal, Consejo Superior de Investigaciones Científicas (CSIC) Madrid Spain

**Keywords:** BRAF^V600E^, LKB1, melanoma, neural crest like, ultraviolet radiation

## Abstract

The mechanisms that work alongside *BRAF* ^*V600E*^ oncogene in melanoma development, in addition to ultraviolet (UV) radiation (UVR), are of great interest. Analysis of human melanoma tumors [data from The Cancer Genome Atlas (TCGA)] revealed that 50% or more of the samples expressed no or low amounts of serine/threonine protein kinase STK11 (also known as LKB1) protein. Here, we report that, in a mouse model, concomitant neonatal *Braf* ^*V600E*^ activation and *Lkb1* tumor suppressor ablation in melanocytes led to full melanoma development. A single postnatal dose of UVB radiation had no effect on melanoma onset in *Lkb1*‐depleted mice compared with *Braf* ^*V600E*^‐irradiated mice, but increased tumor multiplicity. In concordance with these findings and previous reports, *Lkb1‐*null irradiated mice exhibited deficient DNA damage repair (DDR). Histologically, tumors lacking *Lkb1* were enriched in neural‐like tumor morphology. Genetic profiling and gene set enrichment analyses of tumor sample mutated genes indicated that loss of *Lkb1* promoted the selection of altered genes associated with neural differentiation processes. Thus, these results suggest that the loss of *Lkb1* cooperates with *Braf* ^*V600E*^ and UVR, impairing the DDR and increasing melanoma multiplicity and neural‐like dedifferentiation.

## Graphical abstract

UVB radiation cooperates with the *BRAF* ^*V600E*^ oncogene in melanoma development. This study shows that many *BRAF* ^*V600E*^‐mutant human melanomas either lack or show low amounts of LKB1 protein. A UV‐induced mouse model showed that conditional *Lkb1* deletion cooperates with *Braf* ^*V600E*^ in melanoma development, promoting deficient DNA damage repair and the enrichment of a neural‐like differentiation tumor phenotype.

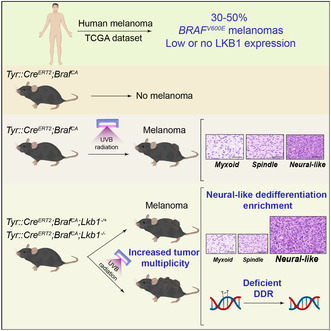

**Abbreviations**
4EBP1: eukaryotic translation initiation factor 4E‐binding protein 1
4OHT: 4‐hydroxytamoxifen
6‐4pps: pyrimidine (6–4) pyrimidone photoproducts
AKT: serine/threonine‐protein kinase Akt
*B*
: *Braf* ^CA^ mouse model
*B;L*
: *Braf* ^CA/+^;*Lkb1* ^−/−^ mouse model
BRAF: serine/threonine‐protein kinase B‐raf
CPD: cyclobutane pyrimidine dimer
DAB: 3,3‐diaminobenzidine tetrahydrochloride
DDR: DNA damage response
DMBA: 7,12‐dimethylbenz[α]anthracene
ERK: extracellular signal‐regulated kinase
FFPE: formalin‐fixed paraffin embedded
IHC: immunohistochemistry
LKB1: liver kinase B1 homolog
MAPK: mitogen‐activated protein kinase
MEK: meiosis‐specific serine/threonine protein kinase
mTORC1: mammalian target of rapamycin complex 1
mTORC2: mammalian target of rapamycin complex 2
NF1: neurofibromin
OIS: oncogene‐induced senescence
PJS: Peutz–Jeghers syndrome
PTEN: phosphatidylinositol 3,4,5‐trisphosphate 3‐phosphatase and dual‐specificity protein phosphatase
RAF: RAF proto‐oncogenes serine/threonine protein kinase
RAS: GTPase RAS
S6: ribosomal protein S6
STK11: serine/threonine protein kinase STK11
TCGA: The Cancer Genome Atlas
TMB: tumor mutational burden
TRP2: l‐dopachrome tautomerase
UV: ultraviolet
UVB: ultraviolet radiation type B
UVR: ultraviolet radiation
VAF: variant allele frequency

## 1 Introduction

Cutaneous melanoma is the most aggressive form of skin cancer and is responsible for the majority of skin cancer‐related deaths. In melanoma, *BRAF* mutation is the most frequent alteration (50% of melanoma tumors) leading to abnormal activation of the RAS/RAF/MEK/ERK signaling pathway [1, 2, 3]. There are no differences in the frequency of BRAF mutations between benign and tumoral lesions [4], indicating that mutated *BRAF* is not sufficient for melanoma transformation [5]. Multiple studies have identified major host and environmental risk factors for melanoma. The predominant environmental risk factor is exposure to UV radiation (UVR), which is responsible for the generation of DNA mutations that, if not properly repaired, can result in genomic instability and, consequently, tumorigenesis [6, 7]. The cooperation of UVR and *BRAF* mutations in promoting malignant melanoma has been previously described in different contexts [8, 9]. In addition to UVR, the expression of BRAF^V600E^ combined with the silencing of the tumor suppressor PTEN [10] or the downregulation of the tumor suppressor NF1 [11] induces melanoma development and progression.

Serine threonine kinase 11 (*STK11*), also known as LKB1, is a ubiquitously expressed and evolutionarily conserved serine/threonine kinase identified as a tumor suppressor. It is involved in a number of biological processes, including DDR [12, 13, 14, 15]. *LKB1* has been found to be mutated in several tumor types, such as lung cancer [16, 17], cervical cancer [18], pancreatic cancer [19, 20], breast cancer [21], and malignant melanoma [22, 23]. In fact, Peutz–Jeghers syndrome (PJS), which results from germline mutations in *LKB1*, is characterized by an increased probability of developing cancer [24, 25]. Several studies have shown that cancer development and progression can be promoted in the absence of *LKB1* even in haploinsufficiency conditions [12]. In the case of melanoma, *Lkb1* inactivation facilitates the expansion of pro‐metastatic melanoma cell subpopulations upon RAS pathway activation [26]. In addition, LKB1 inactivation synergized with *BRAF* ^*V600E*^, promoting cell transformation [27]. Moreover, *Lkb1* deficiency sensitized mice to DMBA‐induced skin and lung squamous cell carcinomas [28] and increased the progression of lung adenomas to carcinomas [29]. Additionally, LKB1 plays a crucial role in the regulation of neural crest cell migration and differentiation during embryonic development, where dysregulation of LKB1 function can lead to defects in neural crest cell development and contribute to various developmental abnormalities [30].

Melanoma tumors are characterized by a high mutational burden resulting from exposure to UVR. *LKB1* loss contributes to tumor development and progression in several tumor types, including *BRAF*‐mutant melanoma, under different genetic conditions. Given that LKB1 plays a relevant role in DDR [12] and neural crest cell differentiation [30], we investigated the contributions of *Lkb1* loss to UVR‐induced melanoma development and progression in a *Braf* ^*V600E*^‐mutant animal model. Our data show that a substantial fraction of *BRAF*‐mutated human melanomas either do not express or express low amounts of LKB1. *In vivo* modeling of UV‐induced *Braf* ^*V600E*^‐mutated melanoma showed that loss of *Lkb1* cooperated with *Braf* ^*V600E*^ and UVR, impairing DDR and increasing melanoma multiplicity and dedifferentiation.

## 2 Materials and methods

### 2.1 Mouse model

All mice were cared for and maintained in accordance with animal welfare regulations under an approved protocol by the Institutional Animal Care and Use Committee at Vall d'Hebron Institut de Recerca (VHIR) and Biomedical Research Park of Barcelona (PRBB). *Braf* ^*CA/CA*^ strain has been previously described [10, 31]. Tyr::*Cre* ^ERT2^; *Lkb1* ^flox/flox^ mice were obtained from Marcus Bosenberg (Yale University, New Heaven, USA). Original Tyr::*Cre* ^ERT2^ mice were from Lynda Chin (Dana Farber, Boston, USA). We crossed the *Tyr::Cre* ^*ERT2*^ *; Lkb1* ^*flox/flox*^ strain with *Braf* ^*CA*/CA^ mice and generated their Mendelian offspring in a mixed genetic background. The *Tyr::Cre* ^*ERT2*^;*Braf* ^*CA/CA*^;*Lkb1* ^*F/F*^ strain was previously described [29]. Both sexes were used for the experiments. All animals were housed and cared for in an SPF‐grade animal facility, under the supervision of the Biomedical Research Park of Barcelona (Prbb) specialized animal facility personnel. All the experiments and studies were performed according to a protocol approved by the Institutional Animal Care and Use Committee at Biomedical Research Park of Barcelona (Prbb) (CEEA: JMC‐10‐1309P2).

#### 2.1.1 Melanoma development *in vivo*

Mice were treated topically on postnatal Days 2.5 and 3.5 with 100 μL of an acetone solution containing 5 mg·mL^−1^ 4‐hydroxytamoxifen (4OHT) (Sigma, St. Louis, MO, USA). Neonatal mice were irradiated on postnatal Day 3.5 as previously described [32]. Tumor development data were analyzed by Kaplan–Meier survival analysis.

### 2.2 Human melanoma samples

All pseudonymized human melanoma tissue samples were provided by the Vall d'Hebron Research Hospital under the National Research Ethics Service approved study number PR(AG)59‐2009. All study methodologies conformed to the standards set by the Declaration of Helsinki. A written informed consent was provided by all patients.

### 2.3 Immunohistochemistry (IHC)

For IHC, 4 μm sections of formalin‐fixed paraffin‐embedded skin or tumor samples were stained following the manufacturer's protocol. The samples were developed by using either secondary antibodies linked to horseradish peroxidase with the UltraView™ Universal DAB Detection Kit (Ventana Medical System; Roche, Tucson, AZ, USA) or secondary antibodies linked to fluorophores. Staining was performed either manually or using the automated immunostainer Beckmarck XT (Ventana Medical Systems). For the samples processed manually, antigen retrieval was performed using a target retrieval solution at pH 6.0 or pH 9.0 according to antibody protocol recommendations (Agilent, Santa Clara, CA, USA). The samples were scanned (panoramic slide digital scanner) and evaluated by two independent pathologists (using 3DHistech Software: Budapest, Öv u. 3., Hungary). LKB1 (#13031; 1 : 250), p‐AKT^S473^ (#4060; 1 : 100), p‐S6^S235/236^ (#2211; 1 : 500), and p‐4EBP1^T37/46^ (#2855; 1 : 1000) antibodies were obtained from Cell Signaling (Danvers, MA, USA). A Cre antibody (NB100‐56135; 1 : 1000) was obtained from Novus Biologicals (Littleton, CO, USA). BRAF^V600E^ (#760‐5095) was purchased from Ventana Medical System. TRP2 (Dct, Pep8) was obtained from V. Hearing (National Institute of Health) [33]. Secondary fluorescent antibodies were purchased from Thermo Fisher Scientific (Waltham, MA, USA). For *H*‐score evaluation, the samples were scanned (panoramic slide digital scanner) and evaluated by two independent pathologists (using 3DHistech software). The *H*‐score was calculated according to the following formula: *H*‐score = [(0 × % negative cells) + (1 × % weak positive cells) + (2 × % moderate positive cells) + (3 × % strong positive cells)], with the overall score ranging from 0 (negative) to 300 (100% strong staining).

### 2.4 Dot blot

The back skins of the *Tyr::Cre* ^*ERT2*^ *;Braf* ^*CA/CA*^ *;Lkb1* ^*F/F*^‐untreated and 4OHT‐treated 2.5‐day‐old neonates were collected 20 h and 7 days after treatment with UVR. Genomic DNA was isolated using a DNeasy kit (Qiagen, Venlo, the Netherlands) following the manufacturer's recommendations. A total of 100 ng of DNA was resuspended in 0.5 m NaOH and 10 mm EDTA, denatured, and spotted on a nitrocellulose membrane using Bio‐Dot SF (Bio‐Rad, Hercules, CA, USA). Then, the membrane was heated at 80 °C for 2 h and incubated with primary antibodies against 6–4 photoproducts (6–4 pps), Dewar photoproducts (Dewar pps), and cyclobutane pyrimidine dimers (CPDs), which were purchased from CosmioBio (Carlsbad, CA, USA). Secondary antibodies were obtained from GE Healthcare (Chicago, IL, USA). Bound antibodies were detected by enhanced chemiluminescence (ECL) (GE Healthcare). DNA loading was assessed by reprobing the membrane with radiolabeled mouse genomic DNA with ethidium bromide (Sigma). Quantification of the spots was performed using imagej 1.53a (NIH, Bethesda, MD, USA).

### 2.5 Whole‐exome sequencing

Whole‐exome sequencing (WES) and data analysis were performed at the Genomic Facility of VHIO (Vall d'Hebron Oncology Institute). In brief, DNA genomic libraries were prepared from fresh tissue (tumor and nontumor) and FFPE tumor tissue DNA prior to exome capture (SureSelect XT Mouse All Exon; Agilent). Libraries were sequenced on a HiSeq2000 instrument (Illumina, San Diego, CA, USA) with a mean coverage of 100×. Reads were aligned, and somatic variants were identified by comparison with nontumor samples (VarScan2).

### 2.6 Statistics

Statistical analyses were performed in graphpad prism 9.0 (GraphPad Software Inc., Boston, MA, USA) using a two‐tailed Student's *t* test and Wilcoxon test to compare differences between two groups.

## 3 Results

### 3.1 A substantial fraction of *BRAF* ^*V600E*^‐mutant human melanomas do not express LKB1

**Fig. 1:**
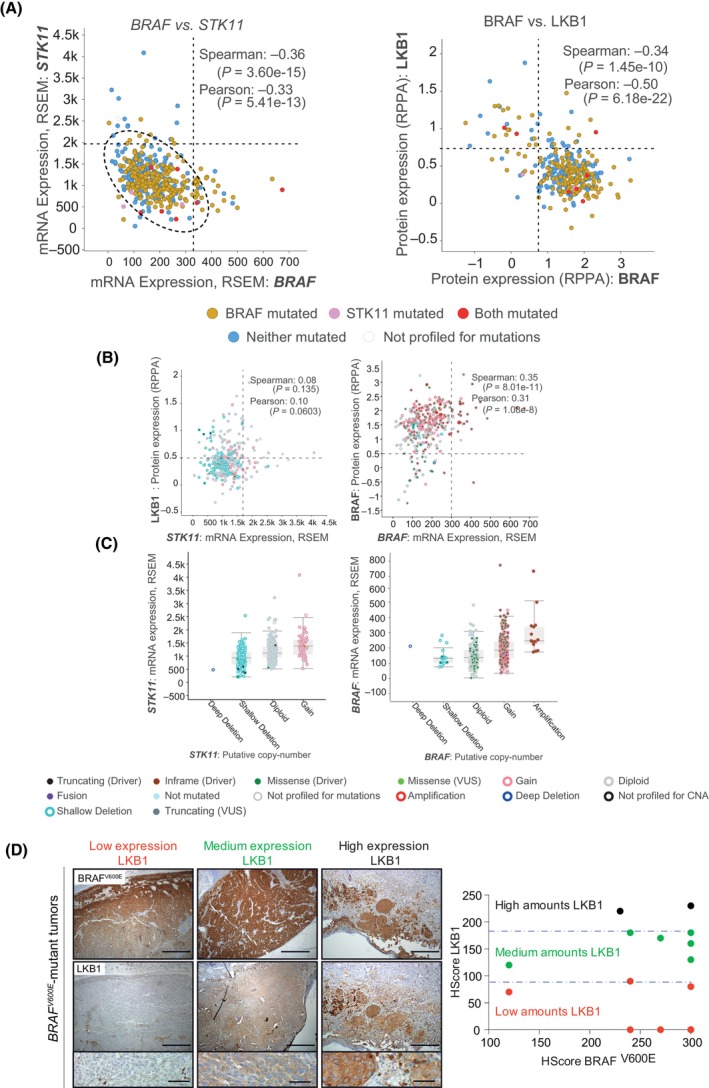
Most BRAF^V600E^‐mutant human melanomas express low or no amounts of LKB1. (A) On the left, correlation between *BRAF* and *STK11* mRNAs in human melanoma samples. On the right, correlation between BRAF and LKB1 protein expression in human melanoma samples. The data were obtained from the cBioPortal database (Skin Cutaneous Melanoma; TCGA, PanCancer Atlas). (B) On the left, correlation between *STK11* mRNA expression and LKB1 protein expression. On the right, there was a correlation between the expression of *BRAF* mRNA and the expression of the BRAF protein. The data were obtained from the cBioPortal database (Skin Cutaneous Melanoma; TCGA, PanCancer Atlas). (C) Analysis of putative *STK11* and *BRAF* genomic copy numbers in relation to mRNA expression. The data were obtained from the cBioPortal database (Skin Cutaneous Melanoma; TCGA, PanCancer Atlas). Bars represent SD. (D) LKB1 immunostaining of BRAF^V600E^ human melanomas. Representative images of different tumor samples expressing low, medium, and high LKB1 levels are shown. On the right, a graphic representation of the LKB1 HScore against the BRAF^V600E^ HScore is shown. The scale bars correspond to 500 and 50 μm.

Loss of *PTEN* and *NF1* tumor suppressors, as well as UVR, cooperates with *BRAF* ^*V600E*^ in melanoma development and progression [10, 11]. In the case of *Lkb1*, it was suggested that its inactivation abrogated *Braf* ^*V600E*^‐induced cell growth arrest [27]. Furthermore, loss of *Lkb1* impaired UV‐induced DDR, leading to genetic instability and the development of skin tumors [12]. To further analyze the role of the tumor suppressor LKB1 in melanomas harboring the *BRAF* ^*V600E*^ mutation, we analyzed the expression and mutational status of *LKB1* in 448 human samples obtained from the TCGA database (Skin Cutaneous Melanoma; TCGA, PanCancer Atlas). The data analysis showed a tendency for a negative correlation between *LKB1* (*STK11*) and *BRAF* mRNA expression, where the absolute number of *LKB1* transcripts was greater than that of *BRAF* mRNA (Fig. 1A). In contrast, most of the tumors, including BRAF‐mutated samples, expressed low amounts of LKB1, while the BRAF protein was highly expressed (Fig. 1A). The correlation between the respective amounts of *LKB1 or BRAF* mRNA and protein supported this observation (Fig. 1B). Similarly, analysis of the putative copy numbers of the *LKB1* and *BRAF* genes revealed that *LKB1* genetic modifications were related to shallow deletions, while *BRAF* genetic alterations principally corresponded to gains and amplifications, including *BRAF* mutations (Fig. 1C). We validated these results by immunohistochemistry in an independent set of human samples (14 human *BRAF* ^*V600E*^‐mutated melanomas). *H*‐score quantification of the samples revealed that while 85% of the samples showed positive staining for BRAF^V600E^, 42% of the samples showed either low or no expression of the LKB1 protein (Fig. 1D). Taken together, these results suggest that a substantial fraction of *BRAF* ^*V600E*^‐mutated melanomas might exhibit LKB1‐dependent functional impairments.

### 3.2 Neonatal loss of *Lkb1* cooperates with *Braf* ^*V600E*^ in melanoma development and increases tumor multiplicity in UVR‐induced melanoma

**Fig. 2:**
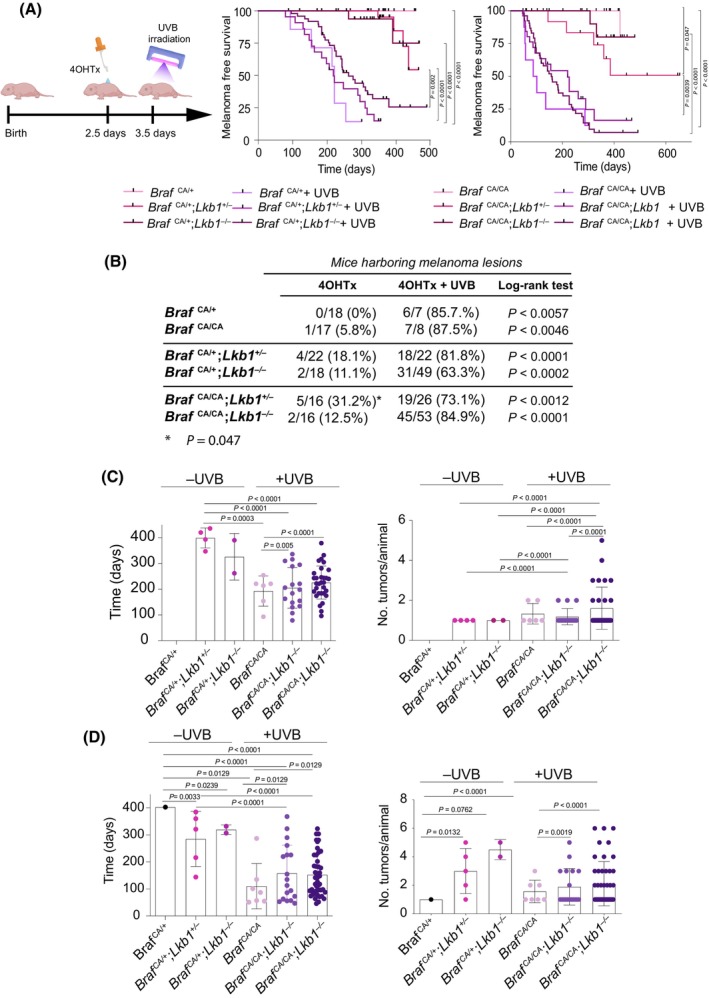
Loss of *Lkb1* cooperates with the *Braf* ^*V600E*^ mutation, promoting melanoma development. (A) Scheme of mouse treatments. Kaplan–Meier survival curve of mice treated with 4‐hydroxytamoxifen (4OHTx) or 4OHTx + ultraviolet radiation (UVR). *Braf* ^*CA/+*^ (*n* = 18)/*Braf* ^*CA/+*^ *; Lkb1* ^*+/−*^ (*n* = 22)/*Braf* ^*CA/+*^ *; Lkb1* ^*−/−*^ (*n* = 18)/*Braf* ^*CA/CA*^ (*n* = 17)/*Braf* ^*CA/CA*^ *; Lkb1* ^*+/−*^ (*n* = 16)/*Braf* ^*CA/CA*^ *; Lkb1* ^*−/−*^ (*n* = 16). (B) Table showing the total number of mice per group and the percentage of mice that developed melanoma tumors in response to either 4OHTx or 4OHTx + UVR treatments. *P* values for the calculated incidences are indicated. (C) Mean tumor onset and tumor multiplicity of *Braf* ^*CA/+*^ mice with or without one or both copies of *Lkb1*. The error bars represent the SDs. The *P* value was calculated by Willcoxon test. (D) Mean tumor onset and tumor multiplicity of *Braf* ^*CA/CA*^ mice with or without one or both copies of *Lkb1*. The error bars represent the SDs. The *P* value was calculated by Willcoxon test.

Epidemiological studies have demonstrated a strong association between UV radiation and melanoma risk, where DNA is the major target of direct or indirect UV‐induced cellular damage. Due to the role of LKB1 in the DDR [12] and the above observation suggesting the lack or low expression of LKB1 in *BRAF* ^*V600E*^‐mutant tumors, we investigated the contribution of *Lkb1* loss to UVR‐induced melanoma development and progression in a *Braf* ^*V600E*^ mutational context. To that end, we used conditional and inducible Tyr::*Cre* ^ERT2^;*Braf* ^*CA*^ mice [10]. *Braf* ^CA^ mice express wild‐type BRAF prior to Cre‐mediated recombination upon 4OH‐tamoxifen treatment, at which time oncogenic *Braf* ^V600E^ is expressed in physiological amounts. To generate the *Tyr*::*Cre* ^ERT2^;*Braf* ^CA^; *Lkb1* ^F/F^ mouse, we crossed the *Tyr*::*Cre* ^ERT2^;*Braf* ^CA^ mouse with the conditional knockout *Lkb1* ^flox/flox^ (*Lkb1* ^*F*/F^) mouse. As previously described, activation of BRAF did not promote melanoma development except in one old homozygous *Braf* ^*CA/CA*^ mouse. However, UVB irradiation promoted melanoma development in 85.7% of *Braf* ^CA/+^ and 87.5% of *Braf* ^CA/CA^ mice. In contrast to previous reports [27], *Lkb1* haploinsufficiency promoted melanoma development via the activation of either one *Braf* ^CA/+^ allele (18.1%) or both *Braf* ^CA/CA^ alleles (31.2%), albeit at late time points, as previously reported (mean onset times of 399 ± 38.9 and 285 ± 45 days, respectively). However, the loss of both *Lkb1* alleles in mice harboring either one *Braf*‐mutant allele (*Braf* ^CA/+^) or both *Braf*‐mutant alleles (*Braf* ^CA/CA^) promoted a slight decrease in the incidence of melanoma, from 18.1% to 11% and from 31.2% to 12.5%, with an onset of 326 ± 90 and 319 ± 17 days, respectively (Fig. 2A). In comparison with that in UVR‐induced melanomas, the tumor incidence in *Braf* ^CA/+^ or *Braf* ^CA/CA^ mice was not further increased upon Lkb1 loss (Fig. 2B). Nevertheless, the loss of the second *Lkb1* allele promoted a slight delay in melanoma development together with an increase in tumor multiplicity (Fig. 2C,D). Overall, the above results showed that the loss of *Lkb1* cooperates with *Braf* ^*V600E*^ in melanocyte transformation, allowing melanoma development. Furthermore, loss of *Lkb1* increased tumor multiplicity, particularly in response to UVR.

### 3.3 UVR‐ and *Lkb1* ^F/F^‐induced melanoma tumors showed activation of mTORC1/2

**Fig. 3:**
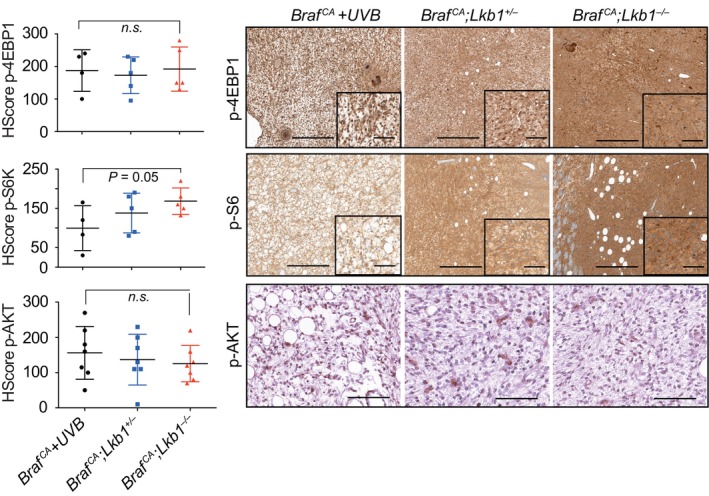
Activation of both mTORC1 and mTORC2 is necessary for *Lkb1* loss‐driven melanoma progression. Representative immunohistochemistry images of tumors from mice with the indicated genetic backgrounds showing p‐4EBP1^T37/46^, p‐S6^S235/236^, and p‐AKT^S473^ staining. Graphs on the left show the HScore for these markers in seven different tumors from each genetic background ± SD. The *P* value was calculated by Student's *t* test. The scale bars correspond to 500 μm.

It has been previously described that *in vivo Braf* ^*V600E*^‐induced melanoma models require the concomitant activation of both mTORC1 and mTORC2/Akt for cell progression to malignancy [27]. The same study suggested that *Lkb1* loss abrogated *Braf* ^*V600E*^‐induced cell cycle arrest but did not lead to melanoma formation. Our data showed that the loss of *Lkb1* (even in haploinsufficiency) allowed the development of *Braf* ^*V600E*^‐mutant melanomas with a low incidence (11.1–31.2%) 10 months after *Braf* ^*V600E*^ activation and *Lkb1* knockout, most likely due to an increase in genomic instability. Since *Lkb1* inactivation leads to mTORC1 activation, we investigated whether these tumors also acquired mTORC2 activation. Independent of the UV irradiation and *Lkb1* status, all analyzed tumors stained positive for surrogate markers of mTORC1 activation, p‐4EBP1^T37/46^, p‐S6^S235/236^, and mTORC2‐mediated phosphorylation of pAKT^S473^ (Fig. 3), suggesting the association of mTORC1/2 activation and *Braf* ^*V600E*^‐driven melanoma. Thus, these results support that the loss of *Lkb1* abrogates *Braf* ^*V600E*^‐induced cell growth arrest, allowing malignant transformation over time and the activation of mTORC1/2.

### 3.4 Loss of *Lkb1* increases tumor heterogeneity and impairs UVR‐induced DDR

**Fig. 4:**
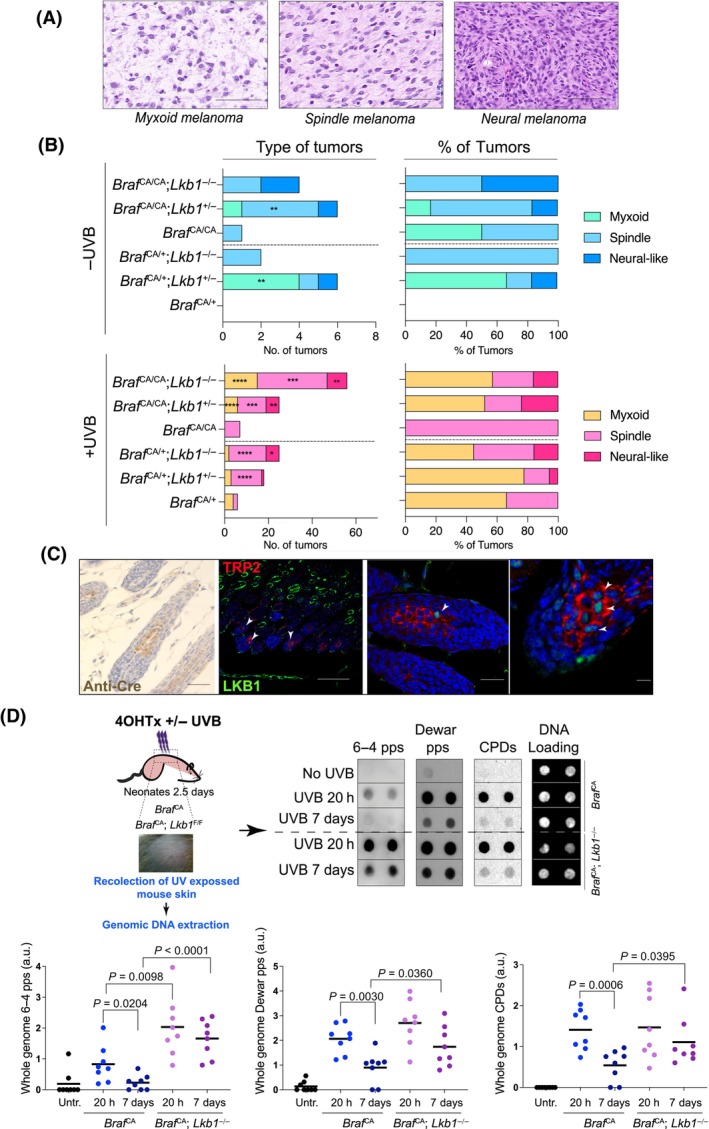
*Lkb1* loss promotes genomic instability and tumor heterogeneity. (A) Representative images of hematoxylin/eosin‐stained tumors (*n* = 45 myxoid; *n* = 96 spindle; *n* = 32 neural‐like) showing the histological melanoma subtypes. Bars represent 500 μm. (B) Graphs representing the number and percentage of the different melanoma subtypes according to the mouse genotype and the administered treatment. *P < 0.05; **P < 0.01; ***P < 0.001; ****P < 0.0001. The *P* value was calculated by Student's *t* test comparing the values between genotypes according to the genetic dose in *Braf*. Number of mice per genotype and condition is indicated in Fig. 2B. In total, *Braf* ^*CA/+*^ (*n* = 25); *Braf* ^*CA/+*^ *; Lkb1* ^*+/−*^ (*n* = 44); *Braf* ^*CA/+*^ *; Lkb1* ^*−/−*^ (*n* = 67); *Braf* ^*CA/CA*^ (*n* = 25); *Braf* ^*CA/CA*^ *; Lkb1* ^*+/−*^ (*n* = 42); *Braf* ^*CA/CA*^ *; Lkb1* ^*−/−*^ (*n* = 69). (C) Representative pictures of mouse skin (*n* = 3 independent mice) stained with anti‐Cre, anti‐LKB1 (green), and the melanocyte marker TRP2 (red). The white arrowheads indicate melanocytes expressing LKB1. Scale bars correspond to 500 μm for the three panels starting from the left and 50 μm for the last panel on the right. (D) Immune Southern blot (identification of DNA modifications by antibodies) of DNA samples from mouse skin 20 h and 7 days after the indicated treatments. The graphs show the quantification of the generated DNA damage. The antibody signal obtained from the different assayed antibodies was normalized with respect to the total DNA amount. The error bars represent the SEMs (*n* = 8). The *P* value was calculated by Student's *t* test.

As previously described [9, 10, 27], melanomas are mainly amelanotic, localized in the dermis or subcutaneous region, with no junctional component, and occasionally display several types of morphologies. We distinguished three major tumor morphologies: melanoma with myxoid features, spindle‐shaped to round plump cell melanomas, and melanomas with neural differentiation (Fig. 4A). *Braf* ^*V600E*^‐mutant tumors derived from UV radiation or *Lkb1* loss were phenotypically heterogeneous (Fig. 4A); nevertheless, we could correlate the predominant type of lesions according to the mouse genotype and treatment (UVR). In absolute number, melanomas with myxoid‐like morphology and spindle melanomas were predominant, and they developed mostly in response to UVR and independently of the absence of *Lkb1*. However, melanomas with neural differentiation were more frequent when *Lkb1* was deleted, with or without UVR, suggesting the existence of tumor morphologies preferentially linked to *Lkb1* dysfunction (Fig. 4B). We analyzed the frequency of different tumor morphologies according to the genetic dose of mutant *Braf*, alone or in combination with the loss of one or both *Lkb1* alleles. Overall, the loss of *Lkb1* increased tumor heterogeneity. UV‐independent myxoid‐like melanomas were absent in either *Braf* ^CA/+^; *Lkb1* ^−/−^ or *Braf* ^CA/CA^; *Lkb1* ^−/−^ and UV‐independent melanomas with neural differentiation were mainly present in mice harboring the activation of two alleles of *Braf* (*Braf* ^CA/CA^) and the deletion of either one or both alleles of *Lkb1* (*Lkb1* ^*+/−*^; *Lkb1* ^*−/−*^) (Fig. 4B). Due to the observed phenotypic heterogeneity of tumor cells and the predominance of certain histological subtypes depending on the *Lkb1* expression status, we analyzed the expression of LKB1 in mouse melanocytes. Interestingly, co‐staining of normal mouse skin (7 days postnatal) for LKB1 and the melanocyte marker TRP2 revealed that only a small fraction of the mouse hair follicle melanocytes stained positive for LKB1 (Fig. 4C), suggesting a possible connection between tumor neural morphology and cellular origin. According to the data, the loss of *Lkb1* increased tumor heterogeneity. *Lkb1* loss impairs UVR‐induced DDR, generating genetic instability. Thus, we analyzed the presence of UVB‐induced DNA damage in mouse skin 20 and 7 days after UVB irradiation. The data revealed that 6–4 photoproducts (6–4 pps), Dewar derivatives, and cyclobutane pyrimidine dimers were still detected at high rates in *Braf* ^CA^;*Lkb1* ^−/−^ mice 7 days after UV irradiation, while they were almost completely repaired in wild‐type mice (Fig. 4D), supporting the role of *Lkb1* in the generation of genomic instability and consequently tumor heterogeneity.

### 3.5 Tumor genetic profiling revealed that *Lkb1* loss was associated with neural differentiation

**Fig. 5:**
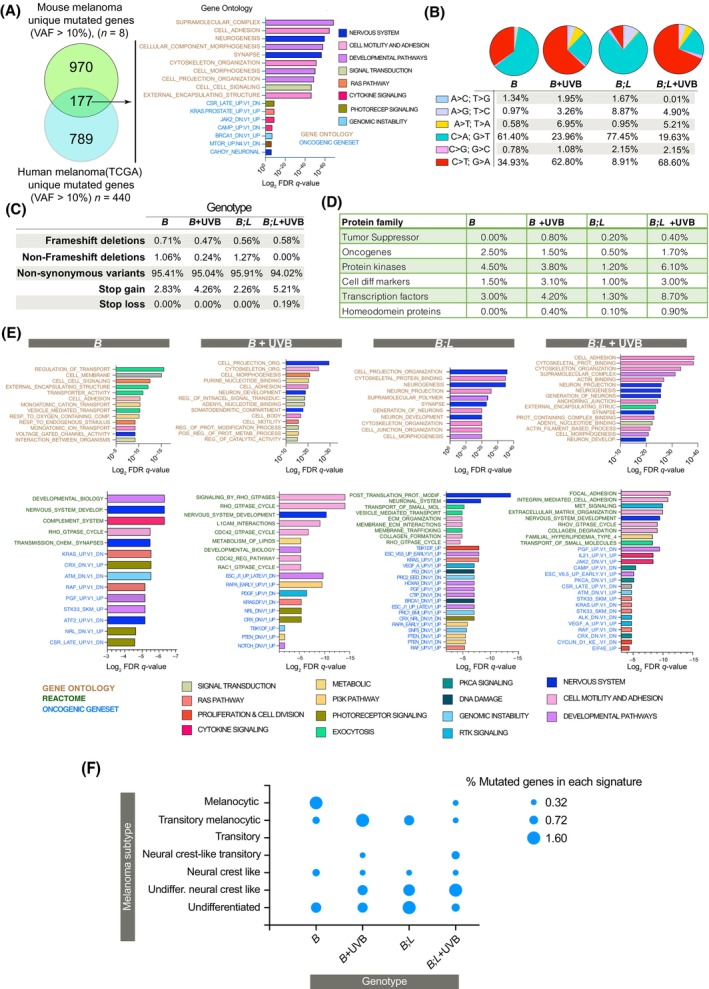
Exome‐sequencing mutational analysis. (A) Venn diagram showing the common unique mutated genes identified in the mouse model (VAF > 10%) and human samples (TCGA, Firehorse database) (VAF > 10%). The biological processes, associated to the identified mutated common genes (Metascape analysis) are shown on the right. (B) Percentage of transitions and transversions detected in each genotype [one *Braf* ^CA/CA^ (*C*), two *Braf* ^CA/+^;*Lkb1* ^−/−^ (*B;L*), and five UVB‐induced tumors (three *Braf* ^CA/+^ and two *Braf* ^CA/+^;*Lkb1* ^−/−^)]. (C) Percentage of different types of genetic alterations found in the indicated genotypes and treatments. (D) Percentage of mutated genes belonging to the indicated groups of proteins. (E) Gene set enrichment analysis of the mutated genes with a variant allele frequency (VAF) > 10% showing the biological processes and pathways altered. (F) Percentage of mutated genes in each subtype of melanoma cells is described in Tsoi et al. [36].

To obtain insight into the mutations contributing to melanocytic transformation by *Lkb1* loss and UVB‐induced melanomagenesis, we performed WES of eight tumors generated from different genetic backgrounds (three spontaneous (one *Braf* ^CA/CA^ (hereafter *B*) and two *Braf* ^CA/+^;*Lkb1* ^−/−^ (thereafter *B;L*)) and five UVB‐induced tumors (three *Braf* ^CA/+^ and two *Braf* ^CA/+^;*Lkb1* ^−/−^)). We identified 1149 unique mutated genes among all analyzed mouse tumor samples (variant allele frequency; VAF > 10%; Table S1). Analysis of the cutaneous melanoma TCGA database (PanCancer) revealed 966 unique genes mutated in human samples (VAF > 10%; Table S1). One hundred and seventy‐seven of these mutated genes, including *Braf* and *Stk11* (*Lkb1*), were identified in mouse and human melanomas. Gene set enrichment analysis revealed that these commonly mutated genes were associated with dysregulated tumor processes, including neural development (dedifferentiation), immune regulation, adhesion, motility, and signal transduction (Fig. 5A and Table S2). Mutational landscape analysis revealed that C>T transitions were the most frequent nucleotide substitutions in *B* + UVB and *B;L* + UVB tumors (62.8% and 68.6%, respectively), while G>T transversions occurred more frequently in *B;L* tumors (77.5%) (Fig. 5B). All tumor types exhibited similar frequencies of alterations (the most frequent nonsynonymous variants), except for stop gains, which were more frequent in UVB‐induced melanomas (Fig. 5C). The identified mutations targeted several protein family subtypes at different frequencies, although *B;L* + UVB tumors showed an increased mutation frequency in protein kinases and transcription factors (Fig. 5D). Then, we investigated which processes were affected by the identified mutated genes according to genotype and treatment. Despite the low number of tumors per group of analysis, genes mutated in UVB‐irradiated tumors (VAF > 20%) were significantly associated with cancer‐related biological processes such as extracellular matrix organization, adhesion, and motility, (*Itga2b*, *Thbs4*, *Tnc*, *Itga1*, *Col6a5*, *Serpine1*, *Ptpn11*, *Cib1*, and *Ift74*), including the activation of RHO signaling (*Lmnb1*, *Myh9*, *Sh3bp1*, *Tuba3a*, *Zap70*, *Tiam2*, *Racgap1*, *Ndufs3*, *Rhobtb1*, *Dnmbp*, *Arhgef6*, *Dock8*, *Cenpi*, *Dock10*, *Fam91a1*, *Pde5a*, *Stard13*, and *Iqgap2*), which is involved in cytoskeletal dynamics and cell movement and is associated with mTORC2 activation [34, 35]. Gene set enrichment analysis of the mutated genes in non‐UVB‐irradiated *Lkb1*‐deficient tumors revealed associations with processes related to neural‐like dedifferentiation (*Neurod6*, *Cttn*, *Dmd*, *Mark2*, *Hsp90ab1*, *Kif5a*, *Lamb2*, *Matn2*, *Thbs4*, *Tulp1*, *Gprin1*, *Cntnap1*, *Camsap2*, *Nrn1*, *Actl6b*, *Bbs4*, *Unc5b*, *Mcf2*, *Ntng2*, *Rnf165*, *Camsap1*, *Lrp4*, *Szt2*, *Gdf7*, *Nes*, *Plec*, and *Fat4*), adhesion, motility, and dysregulation of the RHO signaling pathway (*Atp6ap1*, *Cdc42ep3*, *Arhgap31*, *Mcf2*, *Fmnl3*, *Plekhg6*, *Ptpn13*, *Pkp4*, *Armcx3*, and *Baiap2l2*) (Fig. 5E). Additionally, oncogene gene set analysis revealed that *B;L* tumors harbored mutations in genes associated with p53 (*Crim1*, *Mark2*, *Dmd*, *Amb2*, *Unc13b*, *Msln*, and *Psmb8*), mTOR (*Adgre5*, *Cldn14*, *Irf7*, *Itpka*, *Alox15*, and *Ptprd*), and PTEN (*Fst*, *Nrn1*, *Pdzk1*, *Cldn14*, *Slc6a*, Slc26a4, *Tgfbr3*, *Kcnh7*, and *Lamc3*) activity. Additionally, genes mutated in UVB‐irradiated *B;L* tumors were significantly involved in neural differentiation (i.e., *Col3a1*, *Col5a1*, *Enah*, *Ncam1*, *Sema4d*, *Dpysl3*, *Arhgef7*, *Sptbn4*, *Ptk2b*, *Trpc5*, *Rorb*, *Nyap*, and *Pak4*) and altered genes involved in signal transduction (*Egr4*, *Grid2*, *Fosb*, *Dyrk1b*, *Ror2*, *Prdm1*, *Ptk2b*, *Mapk10*, *Csf3r*, *Hras*, *Stat4*, *Six4*, *Rgs14*, *Grap*, *Pak4 Wnk2*, *Mapkbp1*, and *Arhgef7*) and immune‐modulating processes (*Cav1*, *Ifnb1 Stat4*, and *Il23r*) (Fig. 5E). Furthermore, these results were supported by the percentage of mutated genes belonging to the different signatures defining the melanoma cell subtypes [36] (Fig. 5F and Table S2). Taken together, these results support the cooperation of *Lkb1* loss with *Braf* ^*V600E*^ in melanoma development and its association with morphological neural‐like dedifferentiation.

## 4 Discussion

The signaling pathways that cooperate with MAPK/ERK activation in *BRAF* ^*V600E*^ melanoma are of great interest. Several studies have demonstrated that UVR can cooperate with the *BRAF* ^*V600E*^ mutation, bypassing oncogene‐induced cell cycle arrest and promoting uncontrolled cell proliferation and tumor development [8, 9]. In addition, *Pten* or *Nf1* loss abrogates *Braf* ^*V600E*^‐induced oncogene‐induced senescence (OIS) and leads to *in vivo* melanoma formation and *Lkb1* loss abrogates *Braf* ^*V600E*^‐induced cell growth arrest without full progression to malignancy [10, 11, 37]. Despite the well‐known functions of LKB1 as a tumor suppressor, LKB1 also plays a relevant role in DDR [12, 13, 14, 15], suggesting cooperation among *BRAF* ^*V600E*^ mutations, UVR‐induced DNA damage, and/or *LKB1* loss to promote melanomagenesis.

Initial analysis of human samples from the TCGA database supported previous observations suggesting low expression or inactivation of *LKB1* in melanoma patients [22, 23]. These data also revealed a negative correlation between *BRAF* and *STK11* mRNA and protein expression. While many patients suffer shallow copy number deletions in the STK11 gene, the BRAF locus is subjected to copy number gains or amplifications. However, *STK11* mRNA was more abundant than *BRAF mRNA*, which could be interpreted as a compensatory mechanism at the transcriptional level that did not correlate with the corresponding amount of protein expression. This negative correlation of BRAF and STK11 at the protein level was also observed in our validation subset of *BRAF* ^*V600E*^‐mutated human melanomas, supporting the notion of a lack of expression or low amounts of LKB1 in at least 50% of *BRAF* ^*V600E*^‐mutated samples. Due to this observation and the role of LKB1 in DNA damage repair [12, 13, 14, 15], we investigated the contributions of *Lkb1* loss to UVR‐induced melanoma development and progression in a *Braf* ^*V600E*^ mutational context. In contrast to a previous report [27], *Lkb1* haploinsufficiency cooperated with *Braf* ^*V600E*^, promoting full tumorigenesis, although progression occurred at a longer time than previously reported. Further characterization of these tumors by immunohistochemistry also confirmed the activation of both mTORC1 and mTORC2/Akt pathways for cell progression to malignancy [27]. *Lkb1* loss did not further increase *Braf* ^*V600E*^ UVR‐induced melanoma, probably due to the high tumor penetrance in the model. For unknown reasons currently under investigation, the loss of both *Lkb1* alleles (*Lkb1* ^−/−^) promoted a delay in melanoma development. However, in agreement with the impaired UVB‐induced DNA damage repair and the described role of LKB1 in DDR, the loss of *Lkb1* ^−/−^ contributed to genetic instability, increasing tumor multiplicity, even compared with that in heterozygous mice (*Lkb1* ^*+/−*^). These data also support the acquisition of additional genetic alterations in the absence of UVB radiation, which will facilitate the promotion of full tumorigenesis in *Braf* ^*V600E*^ melanocytes upon *Lkb1* loss. Nevertheless, in irradiated *Braf* ^CA^;*Lkb1* ^−/−^ melanocytes, tumor cells adaptation and evolution (i.e., metabolic rewiring and/or DNA damage repair in the absence of LKB1) to this complex mutational landscape might be one of the causes behind delay in tumor development observed in these mice.

Although three major histological morphologies were identified in most of the samples, melanomas with neural‐like differentiation were more frequent upon *Lkb1* deletion. Tumor morphological heterogeneity increases upon *Lkb1* loss, coincident with the genetic instability generated by an impaired DDR [12, 13, 38]. It is documented that LKB1 governs the formation and maintenance of several neural crest derivatives, including melanocytes [30, 39]. Additionally, melanoma is known to exhibit phenotypic plasticity and trans‐differentiation along vascular and neural lineages [40]. The enrichment in neural‐like morphology in *Lkb1*‐deficient tumors, including UV‐irradiated tumors, suggested a differential melanocyte subtype for tumor origin. This hypothesis was supported by the observed differential expression of LKB1 in melanocytes at 7 days postnatally, and previous reports showing that melanoma can arise from either melanocyte stem cells or differentiated melanocytes depending on the tissue and anatomical site of origin, activation of oncogenic mutations, and/or the or inactivating mutations in tumor suppressors [40]. Our experiments do not distinguish whether LKB1 expression is something transiently related to melanocyte biology, or it is a permanent feature of certain melanocytes within the hair follicle. However, this result suggests that there will be melanocytes particularly affected by the loss of LKB1 (transiently or permanently).

The mutational profiling of mouse tumor samples revealed that 10% of the identified genes were also mutated in human samples, revealing a similar number of unique mutated genes supporting the relevance of the mouse model. In the UVB‐irradiated samples, C‐to‐T transitions accumulated, while in the nonirradiated samples, G‐to‐T transversions accumulated, indicating the participation of alternative mutational mechanisms mostly in the absence of *Lkb1*. Nonsynonymous variants were the predominant type of mutation, and stop gains accumulated, especially in the irradiated samples. There was a slight increase in nonframeshift deletions in the nonirradiated *Lkb1*‐null samples, which also revealed differences in the frequencies of mutated genes in the protein families. The guanine base (G) in genomic DNA is highly susceptible to oxidative stress due to having the lowest oxidation potential. Therefore, G·C → T·A and G·C → C·G transversion mutations frequently occur under oxidative conditions [41]. *LKB1* deficiency increases the sensitivity of cells to radiation‐induced carcinogenesis and ROS, resulting in excessive DNA oxidation and mutation [13, 42], affecting genomic stability in several ways, and contributing to cancer development [43]. In this matter, *Lkb1* inactivation, particularly in melanocytes, where melanin production yields high amounts of hydrogen peroxide, would contribute to DNA damage reflected in the type of point mutations identified in *Lkb1* null tumors. In fact, we have observed that *Lkb1* ^−/−^‐deficient tumors, independently of UVB radiation, harbored an average of fourfold more mutations than *Braf* ^*CA*^ tumors (data not showed). This piece of data agrees with the elevated tumor mutational burden (TMB) observed in NSCLCs from nonsmokers and mouse models upon *LKB1* deficiency [44].

The comprehensive analysis (Metascape) of the mutated gene lists according to the mouse genotype and treatment indicated that *Braf* ^*V600E*^ tumors showed alterations in motility, adhesion, and cell signaling processes. Interestingly, *Lkb1*‐null tumors, independent of UVB radiation status, showed a clear enrichment of neural differentiation‐related processes, supporting the dedifferentiation and predominant neural‐like morphology linked to those tumors. These data are in line with the role of LKB1 signaling in neural development and homeostasis [45], and the development of neural crest cell derivatives such as melanocytes [30]. Furthermore, due to the pleiotropic roles of LKB1 in cancer (i.e., cell viability, invasiveness, and metabolism), the dysregulation of all these distinct aspects will also contribute to mutation landscape selection and malignancy.

## 5 Conclusion

Overall, this study and our previous reports [12, 29, 46, 47] identified the loss of *LKB1* as an important mechanism alongside the *BRAF* ^*V600E*^ mutation in cancer development and progression. The loss of the multitask *LKB1* kinase contributes to melanoma development through dysregulation of multiple processes, including an increase in genomic instability caused by a deficient DDR. Loss of *LKB1* not only makes cells especially vulnerable to UVB radiation and prone to oncogenes and/or tumor suppressors but also promotes melanocyte transformation toward a neural‐like phenotype. Thus, we identified the loss of *LKB1* as a mechanism alongside *BRAF* ^*V600E*^ and UVB that contributes to melanocyte transformation and melanomagenesis.

## Conflict of interest

The authors declare no conflict of interest.

## Author contributions

JAR and KM contributed to conceptualization. KM, PG‐M, RO, YD, EG‐S, BF, JH‐L, IO, JM‐C, and JAR contributed to investigation. JAR, VG‐P, andEM‐C contributed to resources. JAR and VG‐P contributed to funding acquisition. KM, PG‐M, IO, and YD contributed to methodology. JAR, BF, VG‐P, EM‐C, and KM contributed to formal analysis. JAR and KM contributed to writing—review and editing. JAR contributed to supervision.

### Peer review

The peer review history for this article is available at https://www.webofscience.com/api/gateway/wos/peer‐review/10.1002/1878‐0261.13715.

## Supporting information


**Table S1.** List of unique mutated genes in mouse and human samples.

**Table S2.** Melanoma subtype gene signatures.

## Data Availability

The gene alteration data used in this study are publicly available at cBioPortal (Skin Cutaneous Melanoma; TCGA, PanCancer Atlas, 448 samples; https://www.cbioportal.org). The exome‐sequencing datasets reported in this article have been deposited in the National Center for Biotechnology Information (NIH) under accession number PRJNA1073850.
